# Solid-State Nanopore Readout of Programmable DNA and Peptide Nanostructures for Scalable Digital Data Storage

**DOI:** 10.3390/bios15050287

**Published:** 2025-05-03

**Authors:** Lihuan Zhao, Jiajun Wang, Lin-Sheng Wu, Xin Zhao

**Affiliations:** 1 State Key Laboratory of Radio Frequency Heterogeneous Integration, Shanghai Jiao Tong University, Shanghai 200240, China; zhaolihuan@sjtu.edu.cn (L.Z.); wallish@sjtu.edu.cn (L.-S.W.); 2 Guiji Life Sciences Co., Ltd., Suzhou 215000, China; jiajun.wang@outlook.de

**Keywords:** solid-state nanopore, DNA storage, click-chemistry, peptide, DNA nanostructure

## Abstract

DNA information storage holds tremendous potential due to its scalability, long lifespan, and environmental sustainability. The synthesis and reading of complex DNA data structures are of central importance. In this work, we propose new encoding schemes through novel synthesis methods of DNA and peptide nanostructures. Silicon nitride (SiN_x_) solid-state nanopores (ssNPs) are employed as the detection platform to enable scalable and inexpensive reading. This approach is no longer constrained by the limitations of single-base sequencing technologies. Peptide nanostructures are introduced as a data medium via click-chemistry, expanding encoding sources. By integrating a photosensitive PC-linker, this approach endows the data chain with functionalities for encryption and data formatting, enhancing the security and organization of biological information storage. Our study presents a comprehensive framework for data management from data synthesis to post-processing, which includes encryption, decryption, and erasure functionalities.

## 1. Introduction

The reduced lifespan of solid-state chip-based storage media, due to the magnetic attenuation of the hard disks or their fragile mechanical strength, makes them unsuitable for long-term data preservation [1,2]. Digitally storing data using biomolecules, especially DNA, is a technique rapidly gaining attention for the extremely high storage capacity, long lifespan, and excellent stability it allows for [2,3,4,5]. Various encoding methods can be employed when using DNA as a storage medium. For example, utilizing the four natural DNA bases as storage sequences allows for ultra-high-density storage, potentially up to one million times higher than solid-state disks [6]. However, the use of DNA bases as a means of storing information relies heavily on advances in DNA sequencing technologies. Alternatively, DNA nanostructures and proteins can offer unique advantages [7,8,9,10,11] by providing diverse encoding options for each digit, albeit with a slight reduction in encoding capacity, because it takes one or hundreds of bases to represent a bit of information. The flexibility in tailoring DNA or protein nanostructures facilitates signal reading and interpretation [12,13,14].

However, reading DNA nanostructures at the single-molecule level remains challenging because conventional techniques, including spectral analysis and magnetic force, require expensive equipment and complex procedures [15,16]. Recently, the Keyser group proposed using a ~10 nm glass nanopore for reading DNA nanostructures [8,17], as nanopore-based electrical readout is simple and cost-effective. This approach does not require the enzymatic motors necessary for nucleotide sequencing with biological nanopores [18]. Nonetheless, the difficulty of manufacturing nanopipette-based glass nanopores at scale prevents the massively parallel reading necessary for low-cost storage technology. To address these issues, scalable, high-throughput, and modular platforms for encoding and decoding molecular information are critically needed. To this end, we propose using silicon-based solid-state nanopores (ssNPs) that can be fabricated in massive arrays, enabling high-throughput reading. The fabrication of SiN_x_ nanopores has made significant progress, enabling pore sizes ranging from sub-5 nm to several tens of nanometers [19,20,21]. Smaller pore sizes generally offer higher dimensional and spatial resolution, which provides a solid foundation for the high-sensitivity detection of DNA storage structures [22,23]. Compared with biological and glass-based nanopores, silicon-based ssNPs offer mechanical robustness, thermal stability, and compatibility with CMOS integration, which are essential for developing next-generation molecular storage devices [24,25,26].

In this work, DNA hairpin structures with varying sizes of 5 nm, 8 nm, and 10 nm were synthesized and then attached to ΦX174 Virion single-stranded DNA (ssDNA), resulting in nanostructures that carry encoded information. A robust SiN_x_ ssNP sensor of 30 nm diameter effectively reads the encoded data from these nanostructures with a high signal-to-noise ratio. We expand this and combine it with the click-chemistry technique to include peptide-based nanostructures, thus broadening the range of potential encoding sources. Peptides can achieve a more diverse structure and function by designing different amino acid sequences. Such hybrid bio-molecular systems enable multi-dimensional information encoding, combining sequence and conformation, thereby significantly increasing the encoding alphabet. Furthermore, we incorporated a photosensitive linker between the DNA sequence and the nanostructures, enabling data encryption and erasure. This system exemplifies the feasibility of employing DNA and protein nanostructures, in tandem with an ssNP reading approach, for information storage. Together, our approach represents a modular and scalable platform that integrates the versatility of biomolecular encoding with the high-throughput capabilities of semiconductor-based sensing.

## 2. Results

### 2.1. Detection of the ΦX174 Backbone and DNA Hairpins

**Figure 1:**
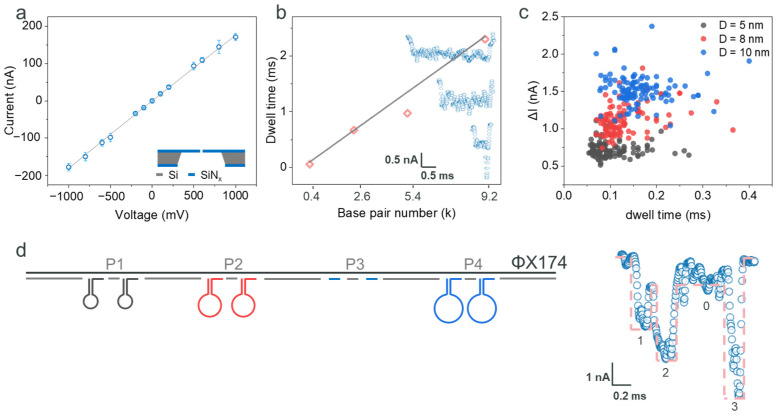
(**a**) Current–voltage characterization of the SiN_x_ ssNP (at least 10 ssNPs were characterized in this study) (illustration of ssNP not to scale). (**b**) The dwell time of different lengths of dsDNA (0.4 kbp, 2.6 kbp, 5.4 kbp, 9.2 kbp). The inset shows the typical current signals during the translocation of these four DNA fragments through the nanopore, from bottom to top: 0.4 kbp, 2.6 kbp, 5.4 kbp, and 9.2 kbp. (**c**) Recording of current and dwell time data of three different DNA hairpins (ring diameter of 5 nm, 8 nm, and 10 nm). (**d**) The composite scheme of the ΦX174 backbone and DNA hairpins, and the current signals of the composited structure detected by the SiN_x_ ssNP platform. DNA hairpins with ring diameters of 5 nm, 8 nm, and 10 nm at positions 1, 2, and 4. All of the electrolyte solution is 2 M LiCl, pH = 8.

We employed SiN_x_-based ssNPs with a diameter of 30 ± 4 nm and a thickness of 20 nm, which were purchased from Norcada Inc., Canada. These ssNPs exhibited a linear I–V relationship and an open pore conductance of 189 ± 18 nS in a 2 M LiCl solution (Figure 1a), consistent with previous studies [9,19]. The nanostructures, functioning as single digital bits, were assembled (annealed) onto a DNA strand backbone. We proposed to use a 5.4 kilobase (kb) ΦX174 single-stranded DNA as the backbone. To verify this, we tested four linear double-stranded DNA (dsDNA) strands with lengths of 0.4 kbp (kilo base pairs), 2.6 kbp, 5.4 kbp, and 9.2 kbp. The characterization of the resistive pulses induced a current amplitude drop of approximately 0.7 nA, and the corresponding typical current signals are shown in the inset of Figure 1b (from bottom to top: 0.4 kbp, 2.6 kbp, 5.4 kbp, and 9.2 kbp). The corresponding dwell times were approximately 0.06 ms, 0.67 ms, 0.97 ms, and 2.3 ms (detailed statistical information is provided in Supporting Information Figure S3). The consistent current drop and the increasing dwell time with longer strand lengths (Figure 1b) suggest that dsDNA translocated through the ssNP linearly. In addition, the 5.4 kb dsDNA was synthesized by the hybridization of ΦX174 single-stranded DNA with 104 complementary oligonucleotides (details in Table S1). Some regions may fail to achieve complete hybridization, leading to DNA folding during translocation. This folding reduces the translocation time, resulting in shorter statistical time data and causing deviation from the linear range. Furthermore, nanoparticles (NPs), specifically 10 nm Fe_3_O_4_ NPs and 20 nm Au NPs (details in Figure S4), were measured using the same ssNP. These results indicate that the sensing resolution of the ssNP could reach below 10 nm and be obstructed by 20 nm nanoparticles. For the nanostructure backbone in this study, a 5.4 kbp sequence inspired by ΦX174 virion was selected. This fully satisfies the temporal and spatial resolution of the nanopores used. This choice also ensures sufficient encoding positions while minimizing the formation of secondary structures, which are more prevalent in longer sequences such as the 9.2 kbp strand (Figure S3c). This careful selection and characterization of the ssNP and DNA sequences enhances the precision and reliability of our nanostructure-based data encoding system.

To maximize the potential of ssNPs in detecting features smaller than 10 nm, we utilized DNA origami to create nanostructures. We synthesized DNA oligonucleotides to form hairpins with diameters of 5 nm, 8 nm, and 10 nm (Figure S5a), and their structural information is shown in Figure S1. The dimensions were estimated based on the axial spacing per base pair in the DNA double helix model (~0.34 nm) [27]. One terminus of the hairpin oligonucleotide is pre-functionalized with an address sequence, which is designed to hybridize with a specific location on the DNA scaffold. The purpose is to enable the targeted positioning of the DNA hairpin, thereby forming a DNA storage structure. Prior to the assembly of the DNA storage structure, a 5 nm DNA hairpin was hybridized with its corresponding address oligonucleotide. A single-stranded address DNA was hybridized with the D = 5 nm DNA hairpin. After hybridization, the DNA hairpin exhibited a reduced mobility in the gel, indicating the effective binding between the hairpin and the address strand, as demonstrated in Figure S5b. These oligonucleotides underwent analysis using ssNP and resistive pulse sensing methods. As shown in Figure 1c, the results indicate a decrease in ionic current for each structure, with the majority of the data points falling within the ranges of 0.55~0.75 nA, 0.85~1.30 nA, and 1.40~1.70 nA, respectively. The dwell times of these hairpins were also recorded, with most values falling within the ranges of 0.05~0.13 ms, 0.09~0.14 ms, and 0.12~0.20 ms, respectively. There was partial overlap in the current and time signals among the three structures. Here, we employed a simple DNA hairpin structure with a single-stranded loop region, which exhibits relatively low rigidity. This reduces the differences between the structures. However, the translocation currents of the three hairpins still showed noticeable distinctions. Interestingly, the outcomes for the 5 nm, 8 nm, and 10 nm hairpins indicate that the 30 nm SiN_x_-based ssNP can detect spatial features as small as 5 nm. This observation is consistent with prior research involving lipid-decorated nanopores [28], NEOtrap technology [29], and exceptionally small nanopores [12,30,31]. The implication is that nanostructures can offer a wider array of encoding options, significantly broadening the scope and adaptability of ssNP-based sensing technologies in molecular biology and nanotechnology.

Here, we developed a method to encode digital information using nanostructure-based digits by binding those DNA hairpins to linearized ΦX174 backbones. We defined the digit address from the 5’ end, selecting four positions (P1, P2, P3, P4), each separated by 1000 bp, as shown in Figure 1d. At each position, we designed two binding sites spaced 50 bp apart, mitigating the risk of hairpin structure binding failure during the annealing process [32]. Furthermore, the sequences assigned to the nanostructure addresses were calculated to achieve encoding orthogonality, with details elaborated in Section 4.2. All other unpaired positions were occupied by complementary oligonucleotides (S1~S104, as listed in Table S1), each approximately 50 nucleotides in length. For coding simplicity, we assigned the following encodings to the nanostructures: ‘1’ for the 5 nm hairpin, ‘2’ for the 8 nm hairpin, and ‘3’ for the 10 nm hairpin. In a proof-of-concept demonstration, we multiplex-encoded the 5 nm, 8 nm, and 10 nm hairpins at positions P1, P2, and P4, respectively. This approach encoded the informative strand as ‘1203’. Utilizing ssNPs and resistive pulse readout techniques, we acquired signature single-molecule signals (as depicted in Figure 1d). The strategic design of polyT sequences at the 3’ and 5’ ends facilitated the efficient capture of informative strands [8]. Analysis of the single-molecule signal (Figure 1d) revealed four distinct current sub-levels: approximately, 2.5 nA, 4 nA, 1 nA, and 5.5 nA. These correspond to the 5 nm hairpin, 8 nm hairpin, intrinsic dsDNA, and 10 nm hairpin, respectively, generating a ‘1203’ code. Furthermore, the dwell time of the informative strand was measured at around 1.2 ms, indicating that the drag force exerted by the binding of the nanostructures is negligible. The dwell time for each sub-level was calculated to be approximately 0.1~0.2 ms, while the presence of two DNA hairpins slightly prolongs the translocation time. However, it does not result in a proportional increase in dwell time. This extended dwell time for each encoded digit is well-suited for state-of-the-art hardware acquisition, demonstrating our encoding strategy’s efficacy.

### 2.2. Encoding with Click Chemistry and Peptides

**Figure 2:**
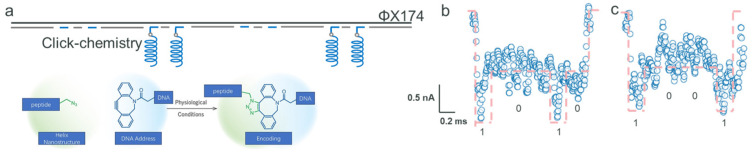
Detection of the digital data structure composed of the ΦX174 strand and peptides through the SiN_x_ ssNP platform. (**a**) The composited scheme of the ΦX174 strand and peptides at positions 2 and 4. The inset below the scheme is the click-chemistry process illustration. (**b**) The current signals of the composite structure ‘1010’. (**c**) The current signals of the composite structure ‘1001’.

Different from the method of annealing address oligonucleotides and nanostructure oligonucleotides, we employed click-chemistry, a technique used to covalently link helical structure peptides (detailed in Supporting Information Figure S2 and Table S1) to the DNA backbone, resulting in a hierarchical storage structure. The peptide, composed of 34 amino acids, has an estimated length of approximately 5 nm based on its helical parameters [33]. We added azide functional groups to the peptides and attached dibenzocyclooctyne (DBCO) groups at the 3’ end of the address oligonucleotides. This configuration enabled the effective attachment of the address oligonucleotides to the DNA backbone (Figure 2a). The formation of a stable covalent bond between the DBCO and azide groups [32] successfully secured the peptides to the DNA backbone. Figure S5c demonstrates the distinction between address bits with and without attached peptides (with a peptide-to-oligonucleotide molar ratio of 1.2:1). Notably, the address bit associated with the peptide exhibits a singular band (~250 bp, suggesting a higher molecular weight), indicating an effective conjugation between the peptide and the address oligonucleotides. Figure S5d illustrates that ssDNA ΦX174 displayed a migration range of around 2~3 kbp, whereas dsDNA ΦX174 had a broader range of 0.3~5 kbp. Considering the extensive use of over 100 staples, there was a possibility of missing staples or address oligonucleotides. Nevertheless, the binding of peptides did not result in noticeable variations in the electrophoresis patterns, even after the omission of address oligonucleotides.

Utilizing the ΦX174 DNA strand as our foundational backbone, we adopted an innovative method by attaching amino acid-based helical nanostructures covalently to predetermined positions, specifically either P1 and P4 or P2 and P4. This technique facilitated the encoding of the informative DNA strand into sequences ‘1001’ and ‘0101’. Specifically, this composite structure can potentially traverse the nanopore beginning from either position 1 or position 4, suggesting that the encoded information generated could potentially correspond to either ‘0101’ or ‘1010’. We noticed fluctuations in current sub-levels during the resistive pulse reading process for single-molecule signal detection. The current decreased to approximately 151.3 nA before rising to around 152 nA, which aligns with the helix nanostructure’s profile (illustrated in Figure 2b). Figure 2c elaborates on the current signal that represents the ‘1001’ sequence. These fluctuations in current sub-levels are indicative of the helix structure, aligning with the stochastic analysis of individual helix peptides, identified by characteristics of 0.53 nA and 0.07 ms (refer to Figure S6). Notably, the ssNP is more sensitive to the spatial attributes of the nanostructure rather than its chemical composition. Consequently, enhancing the nanostructures’ size or altering their structure could markedly improve spatial resolution, thereby increasing the encoding options in terms of both variety and density. Up to this point, we have created multiplexed data sequences ‘1203’, ‘1001’, and ‘0101’, integrating both nucleotide and amino acid elements. Figure S7 showcases the additional current signal data corroborating these results. As shown in Figure S7a,b, there is a slight difference in the dwell time of analytes, which could be attributed to transient fluctuations in electrophoretic force and random interactions or adsorption events between DNA molecules and nanopore surfaces. Alternatively, it may result from subtle variations in nanopore diameter (as depicted in Figure 1a). This progression underlines the adaptability and capacity of our approach in the intricate encoding and interpretation of various biological data at the molecular scale.

### 2.3. Data Encryption

Data encryption is a crucial component of data storage technology. Leveraging the spatial sensitivity of ssNP readout, we propose a method to functionalize encryption by disrupting the translocation process. In the context of this study, the encoding of an encryption digit necessitates an irreversible binding reaction. This approach not only enhances the security of the data storage, but also integrates seamlessly with the ssNP technology, utilizing its inherent properties for innovative encryption solutions.

**Figure 3:**
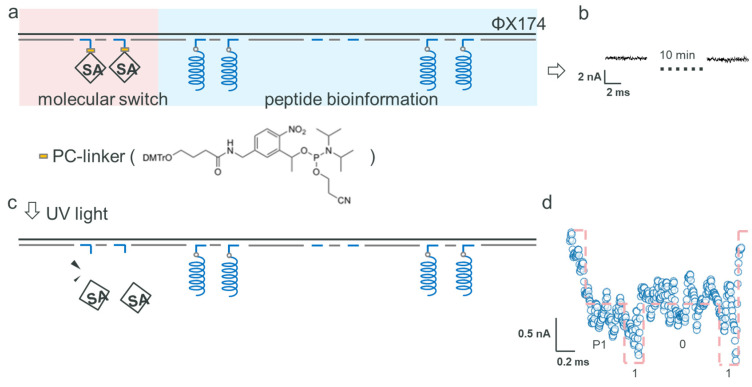
Detection of the digital data structure composed of the ΦX174 backbone strand, SA, and peptides through the SiN_x_ ssNP platform. (**a**) The composite structure of the DNA backbone strand, SA, and peptides, and (**b**) its translocation current signals. (**c**) The result of structure (**a**) after UV illumination. (**d**) The detected current signals.

We harnessed the unique binding affinity between streptavidin (SA) and biotin [32] as a means to enable data encryption. At position P1, we anchored SA to function as the encryption digit. Moreover, we incorporated a photosensitive group at this encryption digit’s site (PC Spacer CE Phosphoramidite, as shown in Figure 3a). This arrangement made it possible to deactivate the encryption digit by removing the SA-biotin conjugate from the backbone under UV light exposure. The ratio of SA molecules to their corresponding binding sites was 1.5:1. For the reading strategy of the helix nanostructure, we affixed nanostructures at positions 2 and 4, creating the ‘P1-101’ pattern, where P1 denotes the encryption digit (illustrated in Figure 3a). During the ssNP reading of the encrypted informative strand, the ssNP’s capture rate remained low for around 10 min, with no clear signals emerging (as observed in Figure 3b). This condition was maintained for over 30 min. Following a 5-min UV (365 nm) exposure in the middle of the reading process, the ssNP started to register effective current signals from the peptide-bound DNA backbone strand. Consequently, this led to the decryption of the code ‘101’, as shown in Figure 3c, disregarding the initial switch bit at position 1 (refer to Figure 3d). This method illustrates the use of SA in hindering the movement of DNA backbones, thereby encrypting biological information. It represents an innovative fusion of biological molecules with photoresponsive components, facilitating controlled and reversible data encryption within DNA-based storage systems.

Furthermore, our observations revealed that solely detecting SA resulted in no fluctuations in the signal, with only the baseline current of the nanopore being noticeable. This suggests that SA alone, without DNA, does not trigger changes in the current within the ssNP. To further investigate, we employed a significantly lower concentration of SA, reduced by two orders of magnitude relative to the molar amount of the corresponding address sites. Given that SA is a tetrameric protein with four biotin-binding sites, multiple DNA strands can potentially bind to a single SA molecule. In this scenario, the high negative charge of DNA may facilitate SA attachment to the DNA backbone, thereby promoting its translocation through the nanopore. Experimentally, we observed signal fluctuations upon reducing the SA concentration. Specifically, a ~6 nA current blockade was recorded, as shown in the left panel of Figure S8. As illustrated in the right panel of Figure S8, after several minutes, nonspecific interactions between the analyte and the nanopore led to irregular signal fluctuations [34]. Different SA concentrations resulted in varied binding behaviors with DNA, and low SA levels were prone to inducing irreversible blockages. In practical applications, the ratio between SA and the number of encrypted address points must be carefully optimized. As such, we have demonstrated that integrating a photosensitive switch is a viable method for encrypting encoded digits on this DNA strand. This technique offers an innovative way to manage access to and the readability of data in DNA-based storage systems.

### 2.4. Data Formatting

**Figure 4:**
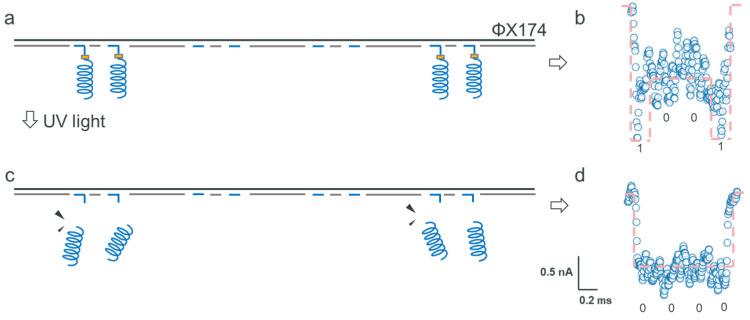
Detection of the digital data structure composed of the ΦX174 backbone strand and peptides through the SiN_x_ ssNP platform. (**a**) The composite of the ΦX174 backbone and peptides at positions 1 and 4 modified by PC-linker, and (**c**) the resulting structure after illumination with UV light. The current signal of the composited structure (**b**) before and (**d**) after UV exposure.

Integrating a photosensitive switch allows for precise encryption at designated positions. This method, when applied to informative digits, also enhances data formatting capabilities. Aligning with this approach, we encoded the helix nanostructure at P1 and P4, creating the ‘1001’ sequence (as depicted in Figure 4a). At the 5’ end of the address oligonucleotides, a photosensitive switch was synthesized, followed by the addition of a DBCO linker. During the ssNP reading phase, we noted a unique resistive pulse signal, characterized by a sub-level around 0.5 nA at the signal’s start and end, signifying the successful decoding of the ‘1001’ sequence (illustrated in Figure 4b). To showcase the data formatting capability, as shown in Figure 4c, we subjected the sample to UV light exposure during the reading process. Consequently, we recorded signals that corresponded only to dsDNA without any biological information (coded as ‘0000’), as shown in Figure 4d. This experiment confirms that employing a photosensitive switch is an effective means to achieve both data encryption and formatting. Contrasting with traditional heating methods that break all hydrogen bonds among nanostructures and address oligonucleotides, as well as backbone strands, the photo-cleavage process is irreversible, eliminating any chance of data retrieval. This technique marks a substantial improvement over standard practices, offering a more secure and accurate method for manipulating data in DNA-based storage systems.

## 3. Conclusions

In summary, this study introduces a scalable and sensitive reading strategy for DNA nanostructures and proteins using SiN_x_ ssNPs with single-molecule resolution. Information is encoded by attaching DNA nanostructures or peptides onto a DNA backbone strand. The use of DNA nanostructures as an information carrier enables the modulation of storage capacity through variations in base number and sequence, thus facilitating multilevel storage capabilities. Click-chemistry is employed to form robust covalent bonds between modified azides and DBCO functional groups. We further integrated SA and a PC linker to serve as a ‘photosensitive switch’, allowing for sophisticated data encryption and decryption strategies. Our study presents a comprehensive framework for DNA storage, encompassing synthesis, reading, encryption, and formatting. Future work will focus on scaling the SiN_x_ nanopore platform to array formats, incorporating a broader range of DNA and protein nanostructures to diversify encoding schemes. We also aim to improve resolution and accuracy to support high-density information identification.

## 4. Materials and Methods

### 4.1. Materials

DNA oligonucleotides were sourced from Guiji Life Sciences Co., Ltd., Suzhou, China, with their specific sequences detailed in Table S1. The 0.4, 2.6, and 9.2 kbp dsDNA samples were also purchased from Guiji Life Sciences Co., Ltd., Suzhou, China. ΦX174 Virion ssDNA, BtsI-v2, and CutSmart buffer were purchased from New England Biolabs. Streptavidin was purchased from Shanghai Macklin Biochemical Technology Co., Ltd., Shanghai, China. The peptide used in this study was purchased from Sangon Biotech (Shanghai) Co., Ltd., Shanghai, China. The PC-linker was purchased from ChemGene. DNA size-selection magnetic beads were purchased from Beyotime Biotechnology (Shanghai, China).

### 4.2. Preparation of DNA Backbone Strand

Initially, we selected the BtsAI enzyme restriction site and engineered a series of eight address bits (Table S1). Subsequently, we applied the UNAfold program to evaluate the orthogonality of these address-bit oligonucleotides [35]. This evaluation revealed that the bits were relatively orthogonal, signifying a notably low mismatch rate.

Step 1. Synthesis of linear ΦX174 ssDNA

The sequence S0 with 25 oligonucleotides was attached to the ΦX174 Virion ssDNA. The solutions include 2 μL ΦX174 ssDNA (1000 ng/μL), 10 μL 10× New England Biolabs cutsmart buffer, 6 μL S0 (10 μM), and 80 μL deionized water.

The solution was mixed and subsequently heated to 95 °C, followed by a cooling ramp to 25 °C over 2 h. Subsequently, 2 μL of BtsI-v2 (10,000 units/mL) was added to the aforementioned mixture and incubated at 37 °C for 1 h. The resulting mixture was then purified using Machery-Nagel NucleoSpin gel. The circular ΦX174 DNA migrated to approximately the 2 kbp position in gel electrophoresis. Upon enzymatic digestion, it was linearized into a ~5.4 kb single-stranded form, which appeared near the 4 kbp position on the gel. The digestion results are presented in Figure S5e.

Step 2. Synthesis of dsDNA structure of ΦX174

After obtaining the linear ΦX174 ssDNA, oligonucleotide sequences spanning from S1 to S104 were employed for hybridization with the ΦX174 ssDNA. The solutions necessary for this procedure include 30 µL linear ΦX174 DNA (5 nM), 13 µL oligonucleotide mix (S1~S104, each oligo 4 μM), 10 µL 100 mM MgCl_2_, 1.5 µL 100 mM Tris-HCl (pH = 8), 10 mM EDTA, 37.5 µL deionized water.

After mixing, the solution was subsequently heated to 95 °C, followed by cooling to 25 °C over 2 h.

We needed to modify different biological groups to address oligonucleotide sequences in different procedures, thus we did not conduct the hybridizations to address oligonucleotide sequences in Step 2.

### 4.3. DNA Hairpins Bind with ΦX174

In this section, six DNA hairpin oligonucleotide sequences with different sizes (D = 5, 8, 10 nm) and two address oligonucleotide sequences (1D5, 2D5, 3D8, 4D8, 7D10, 8D10, 5D0, 6D0, each oligo 4 μM, 1 μL) were mixed and introduced to the synthesis production of Section 4.2. The secondary structure of the DNA was predicted using the NUPACK program [36,37] (Figure S1). The solution was subsequently heated to 55 °C, followed by cooling to 25 °C over 1 h.

Finally, we employed the method of magnetic bead sorting to eliminate any excess staples that did not bond with the backbone.

### 4.4. Peptides Bind with ΦX174

Peptides at positions 2 and 4:

Eight address oligonucleotide sequences (P-3, P-4, P-7, P-8, 1D0, 2D0, 5D0, 6D0, each oligo 4 μM, 1 μL) were mixed and introduced to the synthesis production of Section 4.2. The peptide structure was predicted using the PSIPRED program [38] (Figure S2). The annealing conditions were the same as those of Section 4.3. Subsequently, the peptide was added, in an amount 1.5 times the total molar mass of the address oligonucleotide sequences (P-3, P-4, P-7, P-8). After mixing, the linkage between the polypeptide and the address bits was established.

Peptides at positions 1 and 4:

P-3, P-4, P-7, P-8, 1D0, and 2D0 were replaced by link-A1, link-A2, link-A7, link-A8, 3D0, and 4D0.

The purification method in this stage follows that of Section 4.3.

### 4.5. SA and Peptides Bind with ΦX174

Similarly to in Section 4.4, eight address oligonucleotide sequences (SA-1, SA-2, P-3, P-4, P-7, P-8, 5D0, 6D0, each oligo 4 μM, 1 μL) were mixed and introduced to the synthesis production of Section 4.2, Step 2. The annealing conditions were the same as in Section 4.3. Afterward, SA, which was 1.5 times the molar mass of SA-linkA1 and SA-linkA2, was added. After mixing, SA was linked to the corresponding address oligonucleotide sequences.

A lower amount of SA:

The address oligonucleotide sequences, SA-linkA1 and SA-linkA2, were replaced by SA-1 and SA-2, and the molar mass was 100 times less than the total molar mass of SA-1 and SA-2.

The purification method employed in this final stage aligns with the protocol detailed in Section 4.3.

### 4.6. Measurement Methods

The SSNPs were assembled and fixed using a specialized fixture, along with two Teflon plastic electrolytic cells and two perfluorinated rubber rings, and then the electrolytes in the left and right electrolytic cells could be conducted through the SSNPs. The synthesized DNA product was introduced to the ground electrode side and diluted in a 2 M LiCl solution, resulting in a final concentration of 0.05–0.3 nM. Two AgCl electrodes connected the solutions in both cells to the amplifier (Element srl), while software (Elements Data Reader) was employed for signal recording purposes. The conduction voltage was 600~800 mV, and the amplifier’s sampling frequency was set to 200 kHz with a filter of 15 kHz.

## Data Availability

The data are contained within the article and the Supplementary Materials.

## Supplementary Materials

The following supporting information can be downloaded at https://www.mdpi.com/article/10.3390/bios15050287/s1: Figure S1. Structural diagrams of DNA hairpins with diameters of (a) 5 nm, (b) 8 nm, and (c) 10 nm predicted using the NUPACK program. Figure S2. The peptide structure predicted by the PSIPRED program. Figure S3. Dwell time distribution of dsDNA of (a) 0.4 kbp, (b) 2.6 kbp, (c) 9.2 kbp, and (d) 5.4 kbp in 2 M LiCl, pH = 8 (data points below 0.3 ms were excluded due to their potential origin from the aggregation of ΦX174 and unattached excess staples. Additionally, a small portion of transit times was less than 0.5 ms, raising suspicions about incomplete cutting or agglomeration of ΦX174 circular plasmids. Figure S4. Current signals of (a) 10 nm Fe_3_O_4_ NP and (b) 20 nm AuNP detected using the ssNP platform. Figure S5. (a) Gel (2%) electrophoresis results of three DNA hairpin oligonucleotides. (b) Gel (2%) electrophoresis results of the 5 nm DNA hairpin (D = 5 nm) and the hairpin hybridized with the address strand (50 nt in total, with 25 nt complementary to the hairpin, ccgtttctgataagttgcttgatttTTTTCCTTTCCTTTCCTTTCCTTTC). The annealing transitioned from 55 °C to room temperature over ~2 h, with the hairpin oligonucleotide’s molar quantity at least five times that of the address sequences to ensure their complete hybridization. The DNA hairpin hybridized with the address strand exhibited a reduced mobility in the gel, indicating a shorter migration distance. (c) Gel (2%) electrophoresis results of address oligonucleotides and address binding with peptides. (d) Gel (0.7%) electrophoresis results of ΦX174 ssDNA, dsDNA, and dsDNA with peptides. (e) Gel (0.7%) electrophoresis results of linear ΦX174 ssDNA. Figure S6. Current signals of individual peptides. Figure S7. (a) Effective current signals of positions 2 and 4 bind with peptides, and (b) corresponding repeated experiment. (c) Effective current signals of positions 1 and 4 bind with peptides. Figure S8. Current signals of the composite structure of a lower amount of SA. Table S1: Oligonucleotide and peptide sequences used in this work.
