# Supplementary material for: Solid-State Nanopore Readout of Programmable DNA and Peptide Nanostructures for Scalable Digital Data Storage
Source: Biosensors (Basel). 2025 May 3;15(5):287. doi: 10.3390/bios15050287 (PMC12109865; doi:10.3390/bios15050287)
Supplement: Supplementary file 1 [file biosensors-15-00287-s001.zip › biosensors-3525809-supplementary.pdf]

Solid-State Nanopore Readout of Programmable DNA and Peptide Nanostructures for Scalable Digital Data Storage

Lihuan Zhao [1], Jiajun Wang [2], Lin-Sheng Wu [1] and Xin Zhao [1,*]

[1] State Key Laboratory of Radio Frequency Heterogeneous Integration, Shanghai Jiao Tong University, Shanghai 200240, China; zhaolihuan@sjtu.edu.cn (L.Z.); wallish@sjtu.edu.cn (L.W.)
[2] Guiji Life Sciences Co., Ltd., Suzhou 215000, China; jiajun.wang@outlook.de
* Correspondence: xinzhao@sjtu.edu.cn

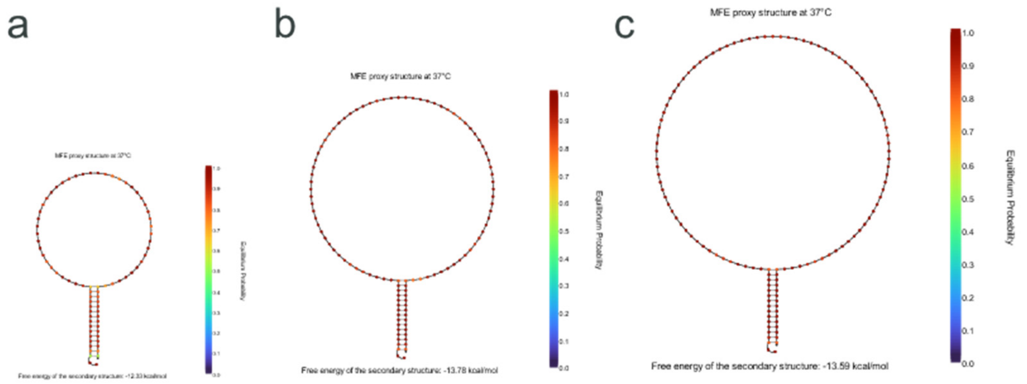

Figure S1. Structural diagrams of DNA hairpins with diameters of (a) 5 nm, (b) 8 nm, and (c) 10 nm predicted by the NUPACK program[36, 37].

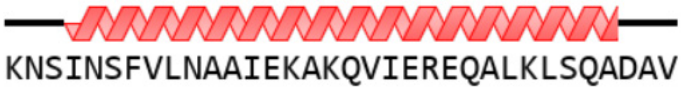

Figure S2. The peptide structure predicted by the PSIPRED program[38].

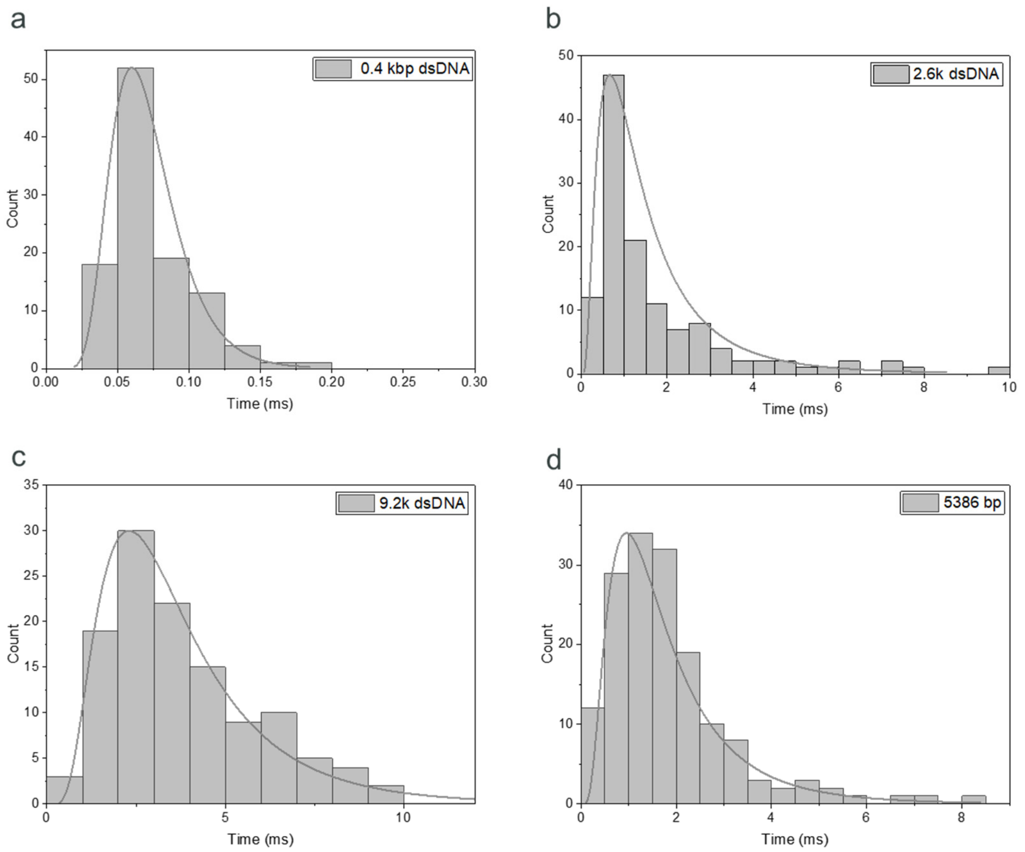

Figure S3. Dwell time distribution of dsDNA of (a) 0.4kbp, (b) 2.6 kbp, and (c) 9.2 kbp (d) 5.4 kbp in 2 M LiCl, pH = 8 (Data points below 0.3 ms were excluded due to their potential origin from the aggregation of ΦX174 and unattached excess staples. Additionally, a small portion of transit times was less than 0.5 ms, raising suspicions about incomplete cutting or agglomeration of ΦX174 circular plasmids.

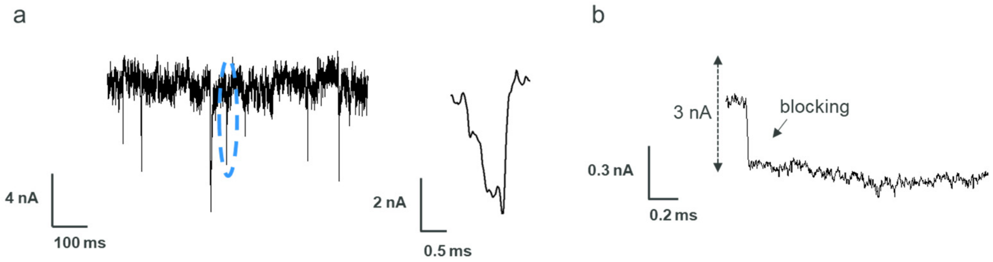

Figure S4. Current signals of (a) 10 nm Fe_3O_4 NP and (b) 20 nm AuNP detected by the ssNP platform.

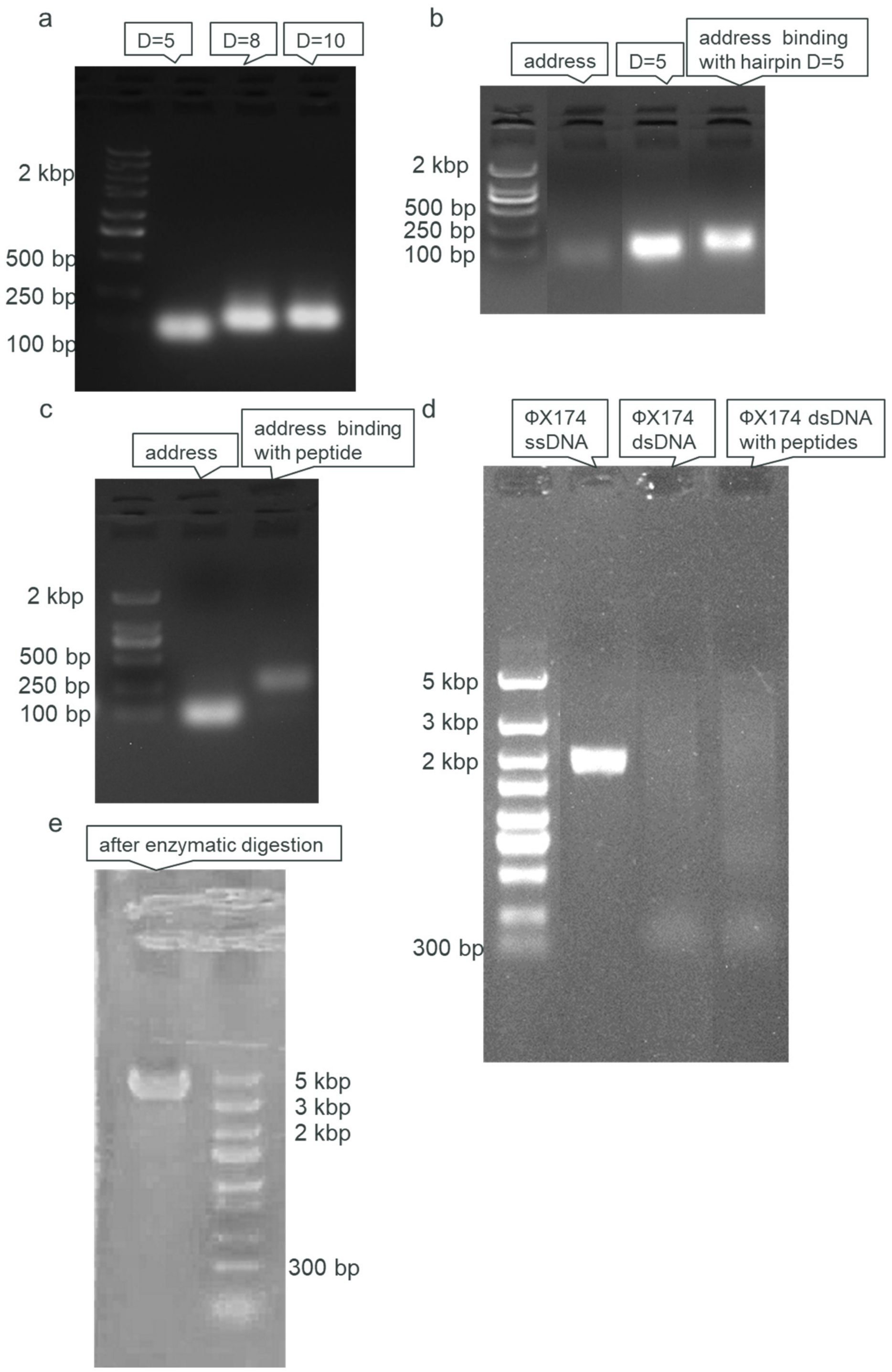

Figure S5. (a) Gel (2%) electrophoresis results of three DNA hairpin oligonucleotides. (b) Gel (2%) electrophoresis results of the 5 nm DNA hairpin (D = 5 nm) and the hairpin hybridized with the address strand (50 nt in total, with 25 nt complementary to the hairpin, ccgtttctgataagttgcttgatttTTTTCCTTTCCTTTCCTTTCCTTTC). The annealing transitioned from 55°C to room temperature over ~2 hours, with the hairpin oligonucleotide's molar quantity at least five times that of the address sequences to ensure their complete hybridization. The DNA hairpin hybridized with the address strand exhibited a reduced mobility in the gel, indicating a shorter migration distance. (c) Gel (2%) electrophoresis results of address oligonucleotides and address binding with peptides. (d) Gel (0.7%) electrophoresis results of ΦX174 ssDNA, dsDNA, and dsDNA with peptides. (e) Gel (0.7%) electrophoresis results of linear ΦX174 ssDNA.

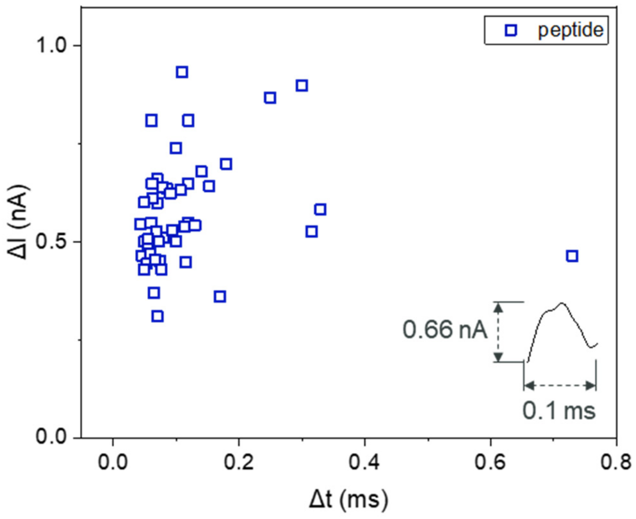

Figure S6. Current signals of individual peptides.

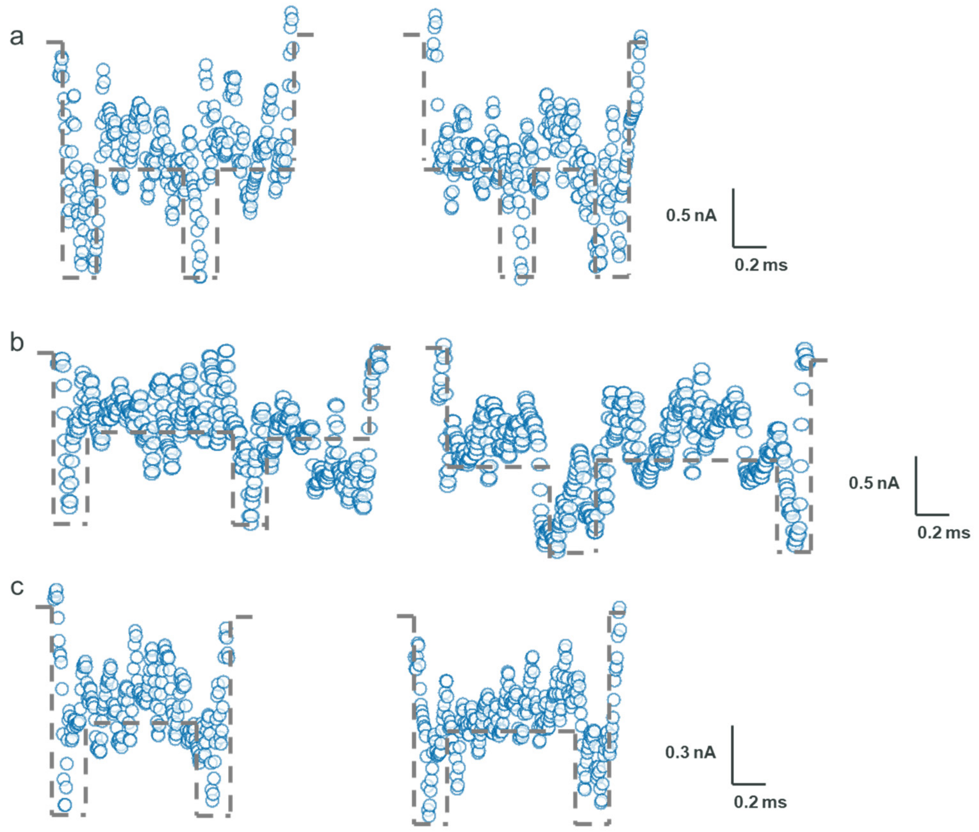

Figure S7. (a) Effective current signals of positions 2 and 4 bind with peptides, and (b) corresponding repeated experiment. (c) Effective current signals of positions 1 and 4 bind with peptides.

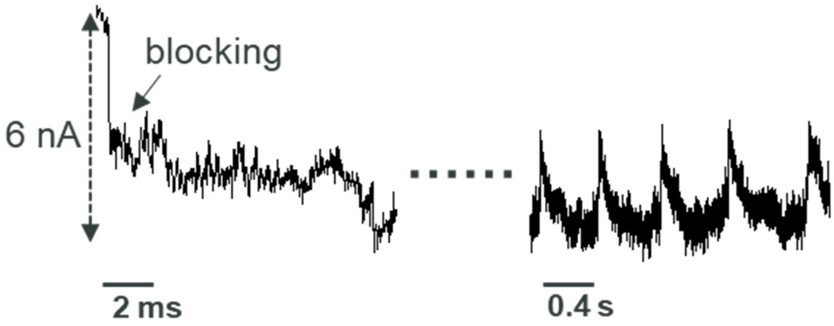

Figure S8. Current signals of the composite structure of a lower amount of SA.

Table S1. Oligonucleotide and peptide sequences used in this work.

Name	Sequence	Lengt h (nt)	Modification method
S0	acctgactattccactgcaacaact	25	
S1	agtggtcggcagattgcgataaacggtcacattaaatttaacctgactatTTTT	54	
S2	gttataacctcacactcaatcttttatcacgaagtcatgattgaatcgcg	50	
S3	cgcttggtcaacccctcagcggcaaaaattaaaatttttaccgcttcggc	50	
S4	taaaagtctgaaacatgattaaactcctaagcagaaaacctaccgcgctt	50	
S5	agaagtgagaaccagcttatcagaaaaaaagtttgaattatggcgagaaa	50	
S6	gatgtagctttaggtgtctgtaaaacaggtgccgaagaagctggagtaac	50	
S7	aaccaccattaccagcattaaccgtcaaactatcaaaatataacgttgac	50	
S8	aacctgattagcggcgttgacagatgtatccatctgaatgcaatgaagaa	50	
S9	gcaaaaaatttagggtcggcatcaaaagcaatatcagcaccaacagaaac	50	
S10	cagtcgggagggtagtcggaaccgaagaagactcaaagcgaaccaaacag	50	

S11	ggtataataaccaccatcatggcgaccatCcaaaggataaacatcatagg	50	
S12	ttattgcccggcgtacggggaaggacgtcaatagtcacacagtccttgac	50	
S13	ggcatttagtagcggtaaagttagaccaaaccatgaaaccaacataaacA	50	
S14	ctggagacaaataatctctttaataacctgattcagcgaaaccaatccgc	50	
S15	agcaataccgccagcaatagcaccaaacataaatcacctcacttaagtgg	50	
S16	ttttgaccgcctccaaacaatttagacatggcgccaccagcaagagcaga	50	
S17	tattgttatcggtagcaagcacatcaccttgaatgccaccggaggcggct	50	
S18	attagagccttgaatggcagatttaataccagcatcacccatgcctacag	50	
S19	gccatagcaccagaaacaaaactagggAcggcctcatcagggttaggaac	50	
S20	cagaagtgccagcctgcaacgtaccttcaagaagtcctttaccagcttta	50	
S21	cacgagtatcctttcctttatcagcggcagacttgccaccaagtccaacc	50	
S22	ccataccagcagaggaagcatcagcaccagcacgctcccaagcattaagc	50	
S23	gtccagttgcattttagtaagctctttttgattctcaaatccggcgtcaa	50	
S24	gcaatctctttttgagtctcattttgcatctcggcaatctctttctgatt	50	
S25	catatacctggtctttcgtattctggcgtgaagtcgccgactgaatgcca	50	
S26	aacgcgagcagtagactccttctgttgataagcaagcatctcattttgtg	50	
S27	tcggaaacctgctgttgcttggaaagattggtgttttccataatagacgc	50	
S28	taaaatactgaccagccgtttgagcttgagtaagcatttggcgcataatc	50	
S29	gtcaacctcagcactaaccttgcgagtcatttctttgatttggtcattgg	50	
S30	ccaatatgagaagagccataccgctgattctgcgtttgctgatgaactaa	50	
S31	caccagaagcagcatcagtgacgacattagaaatatcctttgcagtagcg	50	

S32	gttccaagtatcggcaacagctttatcaataccatgaaaaatatcaacca	50	
S33	ctagacaaattagagccaataccatcagctttaccgtctttccagaaatt	50	
S34	gaggcatgaaaacatacaattgggagggtgtcaatcctgacggttatttc	50	
S35	acattcagaagggtaataagaacgaaccataaaaaagcctccaagatttg	50	
S36	atagcaggtttaagagcctcgatacgctcaaagtcaaaataatcagcgtg	50	
S37	ggaagccaagcattggggattgagaaagagtagaaatgccacaagcctca	50	
S38	aaaagacagaatctcttccaagagcttgatgcggttatccatctgcttat	50	
S39	tggccgtcaacatacatatcaccattatcgaactcaacgccctgcatacg	50	
S40	acttctcagtaacagatacaaactcatcacgaacgtcagaagcagcctta	50	
S41	aatatcaagttgggggagcacattgtagcattgtgccaattcatccatta	50	
S42	aaactgcgtaaccgtcttctcgttctctaaaaaccatttttcgtcccctt	50	
S43	aatacctttctttttggggtaattatactcatcgcgaatatccttaagag	50	
S44	ctacgcgatttcatagtggaggcctccagcaatcttgaacactcatcctt	50	
S45	cagcatgagcctgtcgcattgcattcatcaaacgctgaatagcaaagcct	50	
S46	taatcggtcgtcagccaacgtgagagtgtcaaaaacgataaaccaaccat	50	
S47	atatcacgaaaatagtcacgcaaagcattgggattatcataaaacgcctc	50	
S48	cgtgtgaatcattagccttgcgaccctcggcagcaagaaccatacgacca	50	
S49	gccattagctgtaccatactcaggcacacaaaaatactgatagcagtcgg	50	
S50	ctacctgtaggaagtgtccgcataaagtgcaccgcatggaaatgaagacg	50	
S51	tatttaactggcggcgattgcgtacccgacgaccaaaattagggtcaacg	50	
S52	gcgaactgcgatgggcatactgtaaccataaggccacgtattttgcaagc	50	

S53	gcatcaacaggccacaaccaaccagaacgtgaaaaagcgtcctgcgtgta	50	
S54	catagaaaccaacagccatataactggtagctttaagcggctcaccttta	50	
S55	acctttagcagcaaggtccatatctgactttttgttaacgtatttagcca	50	
S56	agtagcgacagcttggtttttagtgagttgttccattctttagctcctag	50	
S57	ttttcatcccgaagttgcggctcattctgattctgaacagcttcttggga	50	
S58	cttggtaagttggattaagcactccgtggacagatttgtcattgtgagca	50	
S59	tttgcgttctgcttcaatatctggttgaacggcgtcgcgtcgtaacccag	50	
S60	ccaaaacgtcggctacagtaacttttcccagcctcaatctcatctctctt	50	
S61	tatcgaagcgcgcataaatttgagcagatttgtcgtcacaggttgcgccg	50	
S62	tgcgtcatggaagcgataaaactctgcaggttggatacgccaatcatttt	50	
S63	aaacaagcagtagtaattcctgctttatcaagataatttttcgactcatc	50	
S64	agagcttctcgagctgcgcaaggataggtcgaattttctcattttccgcc	50	
S65	gcgtcagtttttgacagaatcgttagttgatggcgaaaggtcgcaaagta	50	
S66	agtccttgacgaacgtgccaagcatattaagccacttctcctcatccaac	50	
S67	gtcaacaagagaatctctaccatgaacaaaatgtgactcatatctaaacc	50	
S68	agtggttgaacagcatcggactcagatagtaatccacgctcttttaaaat	50	
S69	tggaaacgtacggattgttcagtaacttgactcatgatttcttacctatt	50	
S70	aatccaaaacggcagaagcctgaatgagcttaatagaggccaaagcggtc	50	
S71	agcaatccaaactttgttactcgtcagaaaatcgaaatcatcttcggtta	50	
S72	gtaccataaacgcaagcctcaacgcagcgacgagcacgagagcggtcagt	50	
S73	caataaactcaacaggagcaggaaagcgagggtatccTacaaagtccagc	50	

S74	acaggccgtttgaatgttgacgggatgaacataataagcaatgacggcag	50	
S75	tcgacgccattaataatgttttccgtaaattcagcgccttccatgatgag	50	
S76	cgcgtacacgcaaggtaaacgcgaacaattcagcggctttaaccggacgc	50	
S77	tttttgacgcacgttttcttctgcgtcagtaagaacgtcagtgtttcctg	50	
S78	gccagaacgtttttttacctttagacattacatcactccttcTgcacgtaa	50	
S79	ttacgcttgcctttagtacctcgcaacggctgcggacgaccagggcgagc	50	
S80	gcccctgcaattaaaattgttgaccacctacataccaaagacgagcgcct	50	
S81	ggcgccagtttgaatattagacataatttatcctcaagtaaggggccgaa	50	
S82	atctgaccagcaaggaagccaagatgggaaaggtcatgcggcatacgctc	50	
S83	aggagtcgccagcgataaccggagtagttgaaatggtaataagacgacca	50	
S84	tgtctacagtagagtcaatagcaaggccacgacgcaatggagaaagacgg	50	
S85	cattaacaccatccttcatgaacttaatccactgttcaccataaacgtga	50	
S86	agcggcatggtcaatataaccagtagtgttaacagtcgggagaggagtgg	50	
S87	aacaaatgcttagggattttattggtatcagggttaatcgtgccaagaaa	50	
S88	gcatccacggcgctttaaaatagttgttatagatattcaaataaccctga	50	
S89	acgagcatcatcttgattaagctcattagggttagcctcggtacggtcag	50	
S90	ggaagcggagcagtccaaatgtttttgagatggcagcaacggaaaccata	50	
S91	caatagatgtggtagaagtcgtcatttggcgagaaagctcagtctcagga	50	
S92	ttcttggtcagtatgcaaattagcataagcagcttgcagacccataatgt	50	
S93	cctccaaatgaagaaataacatcatggtaacgctgcatgaagtaatcacg	50	
S94	tagagcgcatgacaagtaaaggacggttgtcagcgtcataagaggtttta	50	

S95	taacgacgtttggtcagttccatcaacatcatagccagatgcccagagat	50	
S96	cgcggcacagaatgtttataggtctgttgaacacgaccagaaaactggcc	50	
S97	aacgaacaagcgcaagagtaaacatagtgccatgctcaggaacaaagaaa	50	
S98	agcacctttagcgttaaggtactgaatctctttagtcgcagtaggcggaa	50	
S99	ggcaagttgccatacaaaacagggtcgccagcaatatcggtataagtcaa	50	
S100	tagacgaatcaccagaacggaaaacatccttcatagaaatttcacgcggc	50	
S101	atacgaaggcgcataacgataccactgaccctcagcaatcttaaacttct	50	
S102	ggttcctgaatgaatgggaagccttcaagaaggtgataagcaggagaaac	50	
S103	cataatcatggtggcgaataagtacgcgttcttgcaaatcaccagaaggc	50	
S104	TTTTtccactgcaacaactgaacggactggaaacactggt	40	
1D5	aaatcaagcaacttatcagaaacggTTTTCCTTTCCTTTCCTTCTC ATATCTTCCTATCCTACCTCATATCTTCCTATCCTACC TCATAGGAAAGGAAAGGAA	103	
2D5	tcaggaaatgcagcagcaagataatTTTTCCTTTCCTTTCCTTCTC ATATCTTCCTATCCTACCTCATATCTTCCTATCCTACC TCATAGGAAAGGAAAGGAA	103	
3D8	cggggcggtggtctatagtgttattTTCTCCTCTCCTCTCCTCCTC ATATCTTCCTATCCTACCTCATATCTTCCTATCCTACC TCATATCTTCCTATCCTACCTCATATCTTCCTATAGGA GAGGAGAGGAG	133	
4D8	ggcgttcagcagccagcttgcggcaTTCTCCTCTCCTCTCCTCCT CATATCTTCCTATCCTACCTCATATCTTCCTATCCTAC CTCATATCTTCCTATCCTACCTCATATCTTCCTATAGG AGAGGAGAGGAG	133	
7D10	agagcgccaacggcgtccatctcgaTTCTCCTCTCCTCTCCTCCT CATATCTTCCTATCCTACCTCATATCTTCCTATCCTAC CTCATATCTTCCTATCCTACCTCATATCTTCCTATCCT	148	

	ACCTCATATCTTAGGAGAGGAGAGGAG		
8D10	cgatgagggacataaaaagtaaaaaTTCTCCTCTCCTCTCCTCCTCATATCTTCCTATCCTACCTCATATCTTCCTATCCTACCTCATATCTTCCTATCCTACCTCATATCTTCCTATCCTACCTCATATCTTAGGAGAGGAGAGGAG	148	
1D0	aaatcaagcaacttatcagaaacgg	25	
2D0	aaatcaagcaacttatcagaaacgg	25	
3D0	cggggcggtggtctatagtgttatt	25	
4D0	ggcgttcagcagccagcttgcggca	25	
5D0	agaaatatccgaaagtgttaacttc	25	
6D0	agcagtccacttcgatttaattcgt	25	
SA-1	aaatcaagcaacttatcagaaacggTTTTCCTTTCCTTTC	40	3′ biotin
SA-2	tcaggaaatgcagcagcaagataatTTTTCCTTTCCTTTC	40	3′ biotin
P-3	cggggcggtggtctatagtgttattTTCTCCTCTCCTCTC	40	3′ DBCO
P-4	ggcgttcagcagccagcttgcggcaTTCTCCTCTCCTCTC	40	3′ DBCO
P-7	agagcgccaacggcgtccatctcgaTTCTCCTCTCCTCTC	40	3′ DBCO
P-8	cgatgagggacataaaaagtaaaaaTTCTCCTCTCCTCTC	40	3′ DBCO
SA-linkA1	CTTTCCTTTCCTTTTaaatcaagcaacttatcagaaacgg	40	5′ PC linker & biotin
SA-linkA2	CTTTCCTTTCCTTTTtcaggaaatgcagcagcaagataat	40	5′ PC linker & biotin
link-A1	CTTTCCTTTCCTTTTaaatcaagcaacttatcagaaacgg	40	5′ PC linker & DBCO

link-A2	CTTTCCTTTCCTTTTtcaggaaatgcagcagcaagataat	40	5′ PC linker & DBCO
link-A7	CTCTCCTCTCCTCTTagagcgccaacggcgtccatctcga	40	5′ PC linker & DBCO
link-A8	CTCTCCTCTCCTCTTcgatgagggacataaaaagtaaaaa	40	5′ PC linker & DBCO
Adr-1	AAATCAAGCAACTTATCAGAAACGGtgatccacagactcg	40	
D1-5	TTTTCCTTTCCTTTCCTTCTCATATCTTCCTATCCTACCTCATATCTTCCTATCCTACCTCATAGGAAAGGAAAGGAAcgagtctgtggatca	93	
peptide	K (N3)-SINSFVLNAAIEKAKQVIEREQALKLSQADAV		
